# Pathogenic bacteria and timing of laying

**DOI:** 10.1002/ece3.1473

**Published:** 2015-03-23

**Authors:** Anders Pape Møller, Juan J Soler, Jan Tøttrup Nielsen, Ismael Galván

**Affiliations:** 1 Laboratoire Ecologie, Systematique et Evolution, UMR 8079 CNRS-Université Paris-Sud XI-AgroParisTechBatiment 362, Université Paris-Sud XI, F-91405, Orsay Cedex, France; 2 Estación Experimental de Zonas Áridas (EEZA-CSIC), Carretera de Sacramento s/nE-04120, Almería, Spain; 3 Espedal 4, Tolne, DK-9870, Sindal, Denmark; 4 Departamento de Ecología Evolutiva, Estación Biológica de Doñana – CSIC, c/ Américo Vespucio s/n41092, Sevilla, Spain

**Keywords:** *Accipiter gentilis*, bacteria, goshawk, laying date, reproductive success

## Abstract

Pathogenic bacteria constitute a serious threat to viability of many organisms. Because growth of most bacteria is favored by humid and warm environmental conditions, earlier reproducers in seasonal environments should suffer less from the negative consequences of pathogenic bacteria. These relationships, and the effects on reproductive success, should be particularly prominent in predators because they are frequently exposed to pathogenic microorganisms from sick prey. Here, we presented and tested this hypothesis by sampling bacteria on adult and nestling goshawks *Accipiter gentilis*. We predicted that early breeders and their offspring should have fewer bacteria than those reproducing later during the breeding season. Adult goshawks with a high abundance of *Staphylococcus* on their beak and claws were easier to capture and their laying date was delayed. Moreover, goshawks that laid their eggs later had offspring with more *Staphylococcus* on their beaks and claws. The strength of the association between laying date and bacterial density of nestlings was stronger during the warm spring of 2013, when nestlings suffered from a higher abundance of pathogenic bacteria. Hatching failure and fledging failure were more common in nests with a higher abundance of *Staphylococcus* independently of the number of years occupied, laying date, and age of the female nest owner. These findings imply that timing of reproduction may be under the influence of pathogenic bacteria. Because early breeding goshawks produce more recruits than later breeders, our results suggest a role for pathogenic bacteria in the optimal timing of reproduction.

## Introduction

Timing of reproduction is usually assumed to coincide with the annual peak of food abundance, in particular so in seasonal environments (Perrins 1970, 1991; Drent 2006). However, many individuals lay too late for the seasonal food peak, and such suboptimal timing could be due to food shortage or trade-offs with other fitness components (Perrins 1970, 1991; Drent 2006). Here we present a novel hypothesis for timing of reproduction and its proximate causation suggesting that pathogenic bacteria and other parasites may prevent some females from laying early. If that was the case, we should expect such late breeding females to produce offspring harboring disproportionately many pathogenic bacteria. An alternative pattern of causation is that offspring suffer disproportionately from the negative effects of microorganisms encountered in the nest if parent birds reproduce late in the season when it is warmer and more humid. Bacterial growth is favored by humid and warm environmental conditions (Stolp 1988), and, therefore, hosts should benefit from early reproduction when it is dry and cold. Humid and warm microclimatic conditions characterize the nests of birds, making nests potential incubators of not only birds' eggs, but also of pathogenic bacteria and other microorganisms.

Pathogenic bacteria are potentially important selective agents that can significantly reduce fitness components of their hosts. Several reviews have shown that pathogenic bacteria are an important cause of mortality in many bird species (Hubálek 2004; Benskin et al. 2009). In humans and domestic animals, microorganisms are a common cause of disease or death (e.g., Beaver and Jung 1985; Evans and Brachman 1998; Strauss and Strauss 2002). Pathogenic bacteria may also play an important role in predator–prey interactions by affecting the likelihood of prey being captured, but also by affecting the infection status of predators. Studies of goshawks *Accipiter gentilis* have shown that prey species differ significantly in antimicrobial defenses as reflected by the relative size of the uropygial gland (Møller et al. 2010a). Bird species with a relatively large gland size for their body size experience a reduced probability of falling prey to goshawks relative to the expectation from their relative abundance (Møller et al. 2010a). Furthermore, feathers from prey held an almost threefold greater number of bacteria than feathers from the same species and study sites but lost because of molt (Møller et al. 2012). In contrast, there was no difference in the abundance of fungi on feathers, showing that the findings were specific for bacteria.

Predators often kill prey in poor health status (Packer et al. 2003). For example, prey generally have more parasites than nonprey (Hudson 1986; Temple 1987; Murray et al. 1997; Møller and Nielsen 2007), and they often have infectious diseases that render them susceptible to capture (Møller et al. 2012). Even the mere presence of a predator can lower levels of immunity and subsequently lead to an increase in prevalence and intensity of microparasites in the house sparrow *Passer domesticus* (Navarro et al. 2004). Prey typically have weakened immune defenses compared to nonprey across a range of different species of birds (Møller and Erritzøe 2000), and this is one reason why they disproportionately often die. An elevated level of infectious disease among wild animals (Hudson 1986; Temple 1987; Murray et al. 1997; Møller and Nielsen 2007) has important implications for predators because they do not only benefit from ease of prey capture, but also risk being infected by pathogens carried by their prey. In addition to the acquisition of pathogenic microorganisms from prey (Møller et al. 2010a), predator–prey interactions are far from peaceful and predators continuously risk injury during prey capture and the actual killing. Prey will typically struggle while scratching and pecking the predator to avoid death and to increase the probability of escape (Møller et al. 2011a,b; Møller and Ibáñez-Álamo 2012; García-Longoria Batanete et al. 2014), and the resulting wounds may become infected with microorganisms. Thus, according to this line of reasoning, predators should experience higher risks of microbial infection compared to nonpredators because they more often become wounded.

The objectives of this study were to test four predictions derived from the hypothesis that microorganism–predator interactions affect the timing of breeding in their hosts. Specifically, we predicted that (1) time until capture of adult goshawks as a measure of the effort required for capturing an individual would decrease with increasing abundance of pathogenic bacteria, especially in males that provide the food for females before and during breeding. An infected individual should be less likely to capture prey than a healthy individual, causing the former category to be easier to lure into a baited trap containing easily captured food. This effect should be independent of age, number of years that a territory was occupied, and study year. (2) We predicted that female goshawks with a high abundance of pathogenic bacteria delayed their timing of breeding independent of age, number of years that a territory was occupied, and study year. (3) We predicted that the abundance of pathogenic bacteria on nestlings would increase with delay in laying date and that this effect would be stronger in a year characterized by unfavorable environmental conditions. (4) We predicted that hatching failure and fledging failure would increase in nests with a higher abundance of bacteria, even when controlling statistically for the potentially confounding effects of laying date, clutch size, and number of years occupied. We tested these predictions by relying on extensive samples of bacteria and reproductive data obtained from goshawks during 2012–2013.

The goshawk is the second-most common avian predator of full-grown birds in Europe and North America (Cramp and Simmons 1980; Burfield and van Bommel 2004). It is sexually size-dimorphic forming two size classes of predators potentially exploiting all species of the bird community by largely similar foraging habits (Cramp and Simmons 1980; Kenward 2006). Goshawks prefer pigeons as prey, and the proportion of pigeons in the breeding diet is positively related to annual and lifetime reproductive success (Nielsen and Drachmann 2003). Goshawks breeding in years with a cold spring had a longer reproductive life span, a higher frequency of breeding estimated as the proportion of years with breeding, and a higher number of fledglings produced per successful breeding attempt, resulting in higher lifetime reproductive success (Herfindal et al. 2015). This known relationship between climatic conditions and reproductive success may be partially mediated by pathogenic bacteria being more abundant in warm springs, and thus, the goshawk is a suitable species for testing the hypothetical association between pathogenic bacteria and the time of reproduction.

## Materials and Methods

### Study sites

Jan Tøttrup Nielsen (JTN) systematically visited more than 120 localities with nests of goshawks in northern Vendsyssel (57°10′–57°40′N, 9°50′–10°50′E), Denmark, during April–August 2012–2013, as part of a long-term population study since 1977. See Nielsen and Drachmann (2003) for a detailed description of the study areas. Each nest was visited at least three times during the breeding season. We recorded 72 occupied territories, but not all the sites produced nestlings because of predation, disturbance, and prosecution, hence accounting for the reduction in sample size to the final 48 nests with nestlings.

### Capture of adult goshawks

JTN captured adult goshawks with traps near nest sites during June–July 2012, in total 14 individuals of which nine were females and five males, while only two adult goshawks being captured during June–July 2013 with a similar capture effort and a similar timing of capture attempts relative to the age of nestlings. Adult goshawks were captured in large traps with clap nets placed near the nest site of the goshawks, and to attract the goshawks, we used live domestic pigeons that were placed in a specially protected container with ad libitum food and water. We recorded the time from placing the trap until capture as a measure of the level of hunger in the goshawks, with a longer time interval assumed to reflect a satiated goshawk that was not interested in food. These goshawks were sexed, their wing length measured with a ruler to the nearest mm, their weight recorded on a spring balance to the nearest g, and their beaks and claws sampled for bacteria as described below. All traps were thoroughly cleaned between captures to avoid accumulation of bacteria over time.

### Goshawk reproduction

JTN checked all nest sites of goshawks during March–August 2012–2013. Once having identified nest locations, all nests were visited when nestlings were 6–42 days old (mean (SD) = 22 days (14)), and bacterial swabs were taken as described below. In total, we sampled 102 nestlings from 48 nests. These nestlings also included the offspring of adults that were sampled.

Laying date of the first eggs was determined based on the wing length of nestlings at the first visit to the nest on the assumption that the interval between eggs was 2.5 days and incubation period was 36.5 days (Cramp and Simmons 1980). We included wing length as a continuous covariate reflecting age of nestlings because wing length increases linearly with age across the age classes of nestlings that we have investigated here (Kenward 2006). For exploring the relationship between laying date and probability of recruitment, we considered all nestlings that were ringed during 1979–2013 in our study area and all recruits that were subsequently recovered by the general public and at least reached an age of 3 years.

We estimated hatching success as the number of nestlings divided by clutch size, while fledging success was estimated as the number of fledglings recorded as the number of juveniles at the nest site divided by the number of hatchlings.

The identity of 35 breeding females was determined from individual patterns of color on their primaries and rectrices (Opdam and Müskens 1976; Kühlapfel and Brune 1995; Nielsen and Drachmann 2003). Because all territories were visited annually, the age of reproducing females was estimated from the first year when a female was present in a territory, as yearlings, 2 years old, or at least 3 years old depending on plumage characters (Cramp and Simmons 1980). These age estimates have been corroborated by comparison with age as determined from adults that had been ringed as nestlings.

We estimated the duration of territory occupation as the number of years when a territory had been occupied out of the total number of years 1977–2013, which was 37 years. Thus, territory occupation ranged from 1 to 37 years.

### Bacterial samples

JTN wore latex gloves sterilized with ethanol and took bacterial samples by cleaning first the entire beak with one sterile swab and then the claws with another each slightly wet with sterile sodium phosphate buffer (0.2 mol/L; pH 7.2). The swabs were subsequently preserved individually in an eppendorf tube at 4°C containing the sterile buffer until laboratory analyses during the following 10 days. Samples were sent cooled by courier transport at weekly intervals to ensure that samples were processed at constant intervals following similar storage conditions before, during, and after transport.

The identity of samples was unknown to the laboratory technicians who conducted all bacterial cultivation. In the laboratory, samples were collected from eppendorf tubes after vigorously shaking the eppendorf in vortex for at least three periods of 5 sec. Serial decimal dilutions up to 10^−6^ were cultivated by spreading homogeneously 100 *μ*L of sample (measured with a micropipette) on plates containing four different sterile solid growth media (Scharlau Chemie S.A. Barcelona). We used tryptic soy agar (TSA), a broadly used general medium to grow mesophilic bacteria, and three specific media: Kenner Fecal Agar (KF) for growing bacteria belonging to the genus *Enterococcus*, Vogel–Johnsson Agar (VJ) for bacteria of the genus *Staphylococcus*, and Hecktoen Enteric Agar (HK) for Gram negative bacteria of the family *Enterobacteriaceae*. Plates were incubated at 32°C for 72 h, and afterward, the number of colonies on each plate was counted. Bacterial density was estimated as CFU (colony-forming units). Although 32°C is below the optimum temperature to grow some bacteria, it allows appropriate quantification of all four types of culture media used in this study (see Peralta-Sánchez et al. 2012; Soler et al. 2012a,b). The mean (SD) abundance of the four categories of bacteria was 4.88 (SD = 2.10) for mesophilic bacteria, 0.48 (0.81) for *Enterococcus*, 0.74 (1.11) for *Staphylococcus*, and 3.97 (2.52) for *Enterobacteriaceae*. The Pearson product–moment correlation coefficients between the abundance of different categories of bacteria were all positive ranging from 0.12 to 0.87 with the strongest relationship being between the abundance of mesophilic bacteria and the abundance of *Enterobacteriaceae*. For a more detailed description of agar media and repeatability estimates of intraspecific variation in bacterial growth on eggshells, see Peralta-Sánchez et al. (2010).

### Statistical analyses

We log_10_-transformed all bacterial counts after addition of a constant of one to normalize the data. We tested for consistency in bacterial loads among nestlings within nests in order to estimate repeatability. There was significantly larger among-nest than the within-nest variation in estimates of bacterial density (*F*_21,28_ = 2.22, *P *=* *0.028).

During 2013, we measured the length of beaks and claws to explore whether it predicted bacterial count. None of these measurements were significantly positively related to bacterial counts (four tests for four categories of bacteria for beaks: Pearson *r *<* *0.16, *N *=* *51 nestlings, *P *>* *0.25; four tests for four categories of bacteria for claws: Pearson *r *<* *0.22, *N *=* *51 nestlings, *P *>* *0.12). Consequently, we did not statistically control bacterial counts for length of the sampled beak or claws. Moreover, bacterial loads estimated for claws and beak were closely positively correlated (including adults and nestlings (average values per nest and sex), four tests for four categories of bacteria: Pearson *r *>* *0.27, *N *=* *51 nestlings, *P *<* *0.006) except Enterococci (Pearson *r *=* *0.05, *N *=* *51 nestlings, *P *=* *0.62). For simplicity, we used the mean bacterial counts on beak and claws in the subsequent analyses.

The relationship between bacterial loads of adults and time needed to capture them was analyzed using Kendall rank order correlation and partial rank order correlation because sample sizes were small hence posing problems of violations of assumptions for parametric tests.

Factors explaining variation in bacterial abundance of goshawk nestlings were explored in mixed models with the four bacterial load estimates as response variables. Laying date, female age, duration of territory occupation, and the interaction between laying date and year were included in the models as continuous predictors, sex as a fixed effect, locality as a random effect (to account for differences in number of offspring for each locality), and study year as a random effect. We also made separate models that in addition included the abundance of the other three groups of bacteria as predictors. However, the conclusions remained qualitatively similar, and hence, we only present the former models for brevity. Wing length did not enter as a significant predictor in any of the analyses, and hence, we did not consider this variable in the analyses presented here.

We related hatching success to the abundance of the four groups of bacteria in Kendall rank order correlations. We subsequently related the abundance of *Staphylococcus* to whether there was any failure (a dichotomous variable with hatching failure or no hatching failure), laying date, clutch size, and the abundance of the three other groups of bacteria to estimate the partial effect for the relationship between abundance of bacteria and success. We related fledging success to the abundance of the four categories of bacteria in Kendall rank order correlations. We also related the abundance of *Staphylococcus* to whether there was any failure (a dichotomous variable with fledging failure or no fledging failure), laying date, clutch size, and the abundance of the three other groups of bacteria to estimate the partial effect for the relationship between abundance of bacteria and success.

We quantified the effect of laying date on recruitment probability by estimating the laying date of the eggs of all nests and the date of the eggs that produced recruits that at least reached an age of 3 years. These recruits were from recoveries of ringed fledglings made by the public. We tested whether recruitment was predicted by mean laying date using general linear mixed models (GLMM) with binomial error and logit link function with recruitment at an age of at least 3 years as the response variable, year and locality as random factors, and laying date as a covariate. Finally, we estimated the intensity of directional selection on laying date as the difference in mean laying date for recruits and the mean laying date for all offspring, dividing this difference by the standard deviation of laying date.

Reported values are least square means of transformed values and standard errors (SE). All analyses were made with JMP (SAS 2012).

## Results

### Time till capture of adult goshawks and bacterial loads

**Figure 1:**
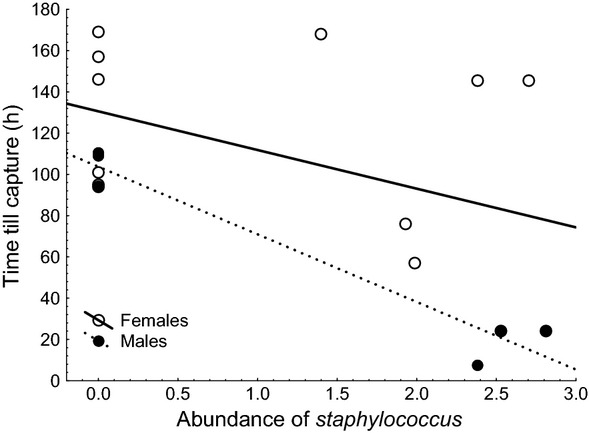
Time till capture of adult male (hatched line) and female (full line) goshawks in relation to the abundance of *Staphylococcus* on their beaks and claws. The lines are the linear regression lines.

We were able to capture 14 breeding adult goshawks during the early nestling period in 2012 and 2 in 2013 with a similar capture effort. This difference is statistically significant (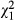 = 7.56, *P *=* *0.006). Time required for catching goshawks ranged from 24 to 169 h (more than 7 days) with a mean of 115 h (SE = 14) in nine females and 8 to 111 h with a mean of 69 h (SE = 5) in five males. Time till capture decreased with increasing abundance of *Staphylococcus* in both females and males (Fig.1; females: Kendall *τ* = −0.24, *P *=* *0.006; males: Kendall *τ* = −0.43, *P *=* *0.003). In females, this effect was also present but did not reach significance for *Enterococcus* (Kendall *τ* = −0.21, *P *=* *0.0022), but did not for mesophilic bacteria or *Enterobacteriaceae* (Kendall *τ* = −0.03 and −0.03, *P *=* *0.68 to 0.70). In males, this effect was not significant for *Enterococcus*, mesophilic bacteria, or *Enterobacteriaceae* (Kendall *τ* = −0.20 to +0.13, *P *=* *0.18 to 0.77). Hence, goshawks of both sexes with more *Staphylococcus* and female goshawks with more *Enterococcus* were easier to catch.

### Fitness components of goshawks and bacterial load

Adult goshawks in our study population during 2012–2013 were on average 5.3 years old (SE = 0.48), range 2–16 years, and *N *=* *43. Their territories had been occupied 23.8 years (SE = 1.56), range 1–37 years, and *N *=* *47. Laying date was on average 15 April (SE = 10.79), range 1–28 April, and *N *=* *48. Clutch size was on average 2.6 eggs (SE = 0.09), range 1–4 eggs, and *N *=* *48. Brood size was on average 2.1 nestlings (SE = 0.13), range 0-4 nestlings, and *N *=* *48. Hatching success was on average 0.83 (SE = 0.04), range 0 to 1, and *N *=* *48, while fledging success was 0.77 (SE = 0.05), range 0 to 1, and *N *=* *48.

**Figure 2:**
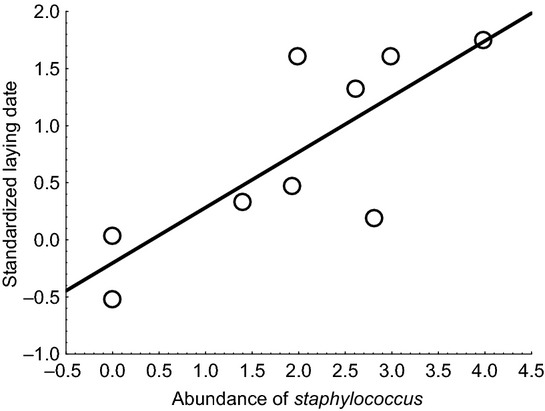
Laying date in goshawks standardized by year in relation to the abundance of *Staphylococcus* on beaks and claws of adult female goshawks. Laying date for each study year was standardized by subtracting the mean and dividing by the standard deviation. The line is the linear regression line.

Next we analyzed the relationship between laying date and abundance of bacteria in the sample of nine captured adult female goshawks. Adult females with more *Staphylococcus* started to breed later than individuals with fewer bacteria (Fig.2; Kendall *τ* = 0.6, *P *=* *0.020). This effect was independent of age of the female and the number of years a breeding territory was occupied. Furthermore, this effect was not significant for *Enterococcus* (Kendall *τ* = 0.50, *P *=* *0.08), mesophilic bacteria (Kendall *τ* = −0.14, *P *=* *0.60), or Enterobacteriaceae (Kendall *τ* = −0.08, *P *=* *0.75). Therefore, adult female goshawks with many *Staphylococcus* started to lay later.

**Figure 3:**
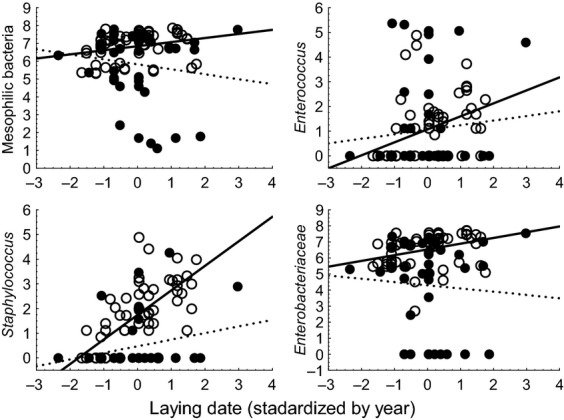
The abundance of four types of bacteria on nestling goshawks in relation to laying date of adult female goshawks standardized by year. Black points and full-drawn lines are for 2012, while open symbols and hatched lines are for 2013. Laying date for each study year was standardized by subtracting the mean and dividing by the standard deviation.

The abundance of *Staphylococcus*, but not of the three other categories of bacteria, on goshawk nestlings was significantly predicted by laying date (Fig.3; Table1 and Table S1). There were no significant effects of age of female, number of years that a site had been occupied, or sex (Table1). There was a highly significant two-way interaction between laying date and year for *Staphylococcus* testing if the effect of laying date differed between the 2 years (Table1). This implied that the increase in abundance of *Staphylococcus* with laying date was stronger in 2012 than in 2013 (Fig.3). There were similar abundances of *Staphylococcus* in the 2 years at the start of the breeding season, while the abundance differed more between years at the end of the breeding season (Fig.3). The interaction between year and laying date was strong for *Staphylococcus* (*F*_1,59_ = 20.54, *P *=* *0.00003, effect size estimated as Pearson *r *=* *0.51), which was not the case for mesophilic bacteria, *Enterococcus*, or Enterobacteriaceae (Table S1). To conclude, goshawk nestlings in nests with late breeding had more *Staphylococcus* than nestlings in early nests, this effect being stronger in 1 year than another.

**Table 1:** GLMM for abundance of *Staphylococcus* in goshawk nestlings in relation to standardized laying date, age of female (years), territory occupation (no. years), locality, and study year. The random effect of year had a variance component of 0.275, 95% CI = −0.509 to 1.060, accounting for 42.25% of the total variance, while the random effect of locality had a variance component of −0.009, 95% CI = −0.088 to 0.069, accounting for 0% of the variance. The model accounted for 56% of the variance. Significant effects are shown in bold

Effect	*F*	df	*P*	Estimate (SE)
Intercept	0.17	1, 2.08	0.72	−0.191 (0.461)
**Laying date**	**29.03**	**1, 29.03**	**<0.0001**	**0.058 (0.012)**
Age of female	0.00	1, 16.86	0.99	−0.0001 (0.0179)
No. years occupied	1.10	1, 15.41	0.31	−0.006 (0.006)
**Laying date × Year**	**19.35**	**1, 37.10**	**<0.0001**	**0.052 (0.012)**

Hatching success was significantly negatively related to the abundance of *Staphyloscoccus* (Kendall *τ* = −0.39, *P *=* *0.0012) and to laying date (Kendall *τ* = −0.27, *P *=* *0.019). The abundance of *Staphylococcus* was higher in nests with some hatching failure compared to nests that were successful (failure: 1.43 (0.32), *N *=* *16, successful: 0.40 (0.15), *N *=* *32; *F*_1,46_ = 11.32, *P *=* *0.0016). That was also the case when including laying date, clutch size, and the abundance of the three other groups of bacteria into the model (partial effect of failure: *F*_1,43_ = 6.92, *P *=* *0.012).

Fledging success was significantly negatively related to the abundance of *Staphylococcus* (Kendall *τ* = −0.40, *P *=* *0.0018) and to laying date (Kendall *τ* = −0.42, *P *=* *0.0003). The abundance of *Staphylococcus* was higher in nests with some fledging failure compared to nests that were all successful (failure: 1.40 (0.28), *N *=* *22, successful: 0.19 (0.09), *N *=* *26; *F*_1,46_ = 19.98, *P *<* *0.0001). That was also the case when including laying date, clutch size, and the abundance of the three other groups of bacteria into the model (partial effect of failure: *F*_1,43_ = 5.95, *P *=* *0.019).

### Laying date and recruitment in goshawks

Laying date of 352 clutches from our study population during 1977–2013 that subsequently recruited at least one offspring was 6.87 April, SD = 5.83 days). This value differed significantly from the expected value of 7.59 April for all 1055 clutches from our study area (one-sample *t*-test, *t*_127_ = −2.31, *P *=* *0.021). The difference between observed and expected value was 0.19 standard deviation units. A GLMM of recruitment with year and locality as random factors and laying date as a covariate revealed a significant effect of laying date (*F*_1,934_* *= 11.92, *P *=* *0.0006, estimate (SE) = −0.011 (0.003)). The random effect of year had a variance component of 0.008, 95% CI = −0.001 to 0.017, accounting for 2.5% of the variance, while the random effect of locality had a variance component of 0.007, 95% CI = −0.002 to 0.016, accounting for 2.2% of the variance. Hence, recruits came from clutches that were laid earlier than expected by chance.

## Discussion

The abundance of *Staphylococcus* was larger on adult goshawks that took shorter time to catch in a trap. Adults with more *Staphylococcus* started to lay their eggs later, and nestlings that hatched from such late eggs had more bacteria of the genus *Staphylococcus* than early-hatched nestlings. In addition, hatching success and fledging success decreased with increasing abundance of *Staphylococcus*. Finally, early-hatched offspring were more likely to recruit to the breeding population. These findings are consistent with the hypothesis that bacteria are correlated with laying date. Obviously, this is a correlational study implying that potentially confounding factors must be considered as we have done, but also that we cannot be certain about causation. We discuss a number of alternative scenarios in an attempt to address these potential problems.

Pathogenic microorganisms have commonly been identified from wild birds (Hubálek and Halouzka 1996; Hubálek 2004), although their consequences for host fitness remain poorly understood. Here we analyzed the relationship between the abundance of four different groups of cultivable bacteria and timing of reproduction in goshawks. Although we relied on estimates from culture rather than molecular analyses, these two types of estimates are sometimes positively related (Stolp 1988; Lee et al. 2013), although not always (Donachie et al. 2007). The four categories of bacteria (mesophilic bacteria, *Enterobacteriaceae*, *Staphylococcus*, and *Enterococcus*) may be opportunistic although some taxa under some circumstances may be pathogenic, while others may have beneficial effects (Krieg and Holt 1984; Houston et al. 1997; Singleton and Harper 1998; Franz et al. 1999; Salyers and Whitt 2002; Cook et al. 2003, 2005; Moreno et al. 2003; Soler et al. 2008, 2010, 2011a,b; Peralta-Sánchez et al. 2010; Møller et al. 2011a,b). We are currently using molecular methods to further assess the effects reported here. However, it seems unlikely that *Staphylococcus* were beneficial to goshawks because higher abundance of *Staphylococcus* in goshawks was associated with a delay in timing of reproduction, reduced probability of recruitment, reduced hatching, and fledging success. It is difficult to imagine alternative scenarios that could account for these negative relationships between the abundance of *Staphylococcus* and these important fitness components.

### Fitness components of goshawks and bacterial load

Goshawks are single-brooded raptors with a steep decline in reproductive success with advancing laying date (Kenward 2006; Møller et al. 2010b). Here we have shown that the abundance of bacteria of the genus *Staphylococcus* both on adults and nestlings was strongly negatively related to laying date. In contrast, there was little or no association between fitness components of goshawks and the abundance of the other categories of bacteria. Hence, we can safely conclude that this is not a ubiquitous response by all bacteria. No other factor (female age, number of years of territory occupation) explained variance in laying date of goshawks. This effect of bacteria was stronger in 1 year than another, and it was stronger in *Staphylococcus* than in the three other categories of bacteria. Bacteria on both goshawk adults and nestlings were particularly abundant in nests with late start of breeding. Two interpretations are possible. First, bacteria may become more abundant as the season progresses because of increasing temperatures. Second, early and late breeding goshawks may differ in phenotypic quality with early breeders being more tolerant to bacteria than late breeders, or early breeders having survived pathogen-mediated selection and hence being older than late breeders. We showed that the association between laying date and bacterial abundance was independent of female age and the number of years that a territory had been occupied. These findings are not consistent with the second possibility. The effect of bacteria on laying date may arise as a consequence of the effect on phenotypic condition of the breeding pair (the female producing the eggs, and food provisioning by the male), or because climatic conditions that affect the risk of bacterial contamination also affect the quality of the territory (such as prey availability, nest quality, and other factors). However, given that territory occupation was not a significant predictor of bacterial load of adults or nestlings, it is difficult to imagine that the effect reported here was mediated by territory quality. Although experimental tests are required for determining to which extent the findings reported here are causal, the detected relationship between abundance of bacteria on nestling raptors and laying date suggests that bacteria mediated the known fitness consequences of late breeding. This interpretation is also consistent with hatching and fledging success in goshawks being negatively related to a high abundance of *Staphylococcus*. Such negative effects of bacteria have recently been documented for several species of birds (Brandl et al. 2014; Benskin et al. 2015).

We used the ease of capture of adult goshawks in response to a baited trap as an index of hunger. We hypothesized that adult goshawks with more bacteria would be easier to catch simply because such individuals would harbor more bacteria. Consistent with this prediction, we found that the abundance of *Staphylococcus* was larger on adult goshawks that took shorter time to catch in a trap. In contrast, there was no similar effect for the three other categories of bacteria, nor were there effects of any other potentially confounding factors. We cannot come of with alternative explanations for this association.

The detected relationship between bacteria and laying date of goshawks is unlikely to only be restricted to the lifestyle of raptors, and thus, bacteria may also affect the optimal timing of reproduction in other species. Species of birds may differ in their exposure to horizontally transmitted microorganisms, and species that reuse nest sites such as hole nesters and colonial breeders are likely to suffer the most from parasitism (Møller and Erritzøe 1996). We believe that nests in general constitute optimal habitats for propagation of bacteria and other microorganisms, mainly because high temperatures and high humidity associated with biological activities may facilitate propagation of microorganisms. This selection pressure is likely to be at the origin of a number of antimicrobial defenses in bird nests (Wimberger 1984; Mennerat et al. 2009; Peralta-Sánchez et al. 2010; Møller et al. 2013).

The bacterial hypothesis for laying date presented here and the Perrins hypothesis suggesting that food availability accounts for optimal timing of reproduction (Perrins 1970, 1991; Drent 2006) differ with respect to the underlying mechanism (Table1). The evidence that we have presented here is consistent with pathogenic bacteria playing a role in determining timing of laying. Most prey species of the goshawk have advanced their timing of reproduction considerably during recent decades due to climate change (Møller et al. 2010b). This also applies to the wood pigeon *Columba palumbus* that is a main prey species of the goshawk, although we emphasize that wood pigeons produce multiple broods during an extended period from April to November (Møller et al. 2010b). However, predators such as the sparrowhawk *Accipiter nisus* (Nielsen and Møller 2006) and the goshawk (J. T. Nielsen and A. P. Møller unpubl. data) have barely changed their timing of reproduction, resulting in mistiming of reproduction by predators relative to the timing of peak food availability. If prey have advanced heir timing of reproduction, while predators have not, this should result in mistiming of reproduction relative to food availability and hence inferior body condition of adult goshawks and increased susceptibility to bacterial infection. In fact, while we captured 12 adult goshawks in the colder and wet spring of 2012, we only captured two adults in 2013 with a similar capture effort. Information on timing of reproduction in prey and goshawks offers an opportunity to investigate the relative importance of food availability and bacteria for timing of breeding in goshawks because temperature and food availability for predators have changed independently.

### Factors explaining variation in bacterial density of goshawks

Nestling raptors like all young birds have naïve immune systems that are not yet fully developed (Starck and Ricklefs 1998), and yet they have to consume food that may be tainted by pathogenic bacteria. Adult raptors often encounter pathogenic microorganisms because such prey are more likely to be captured and hence consumed (Møller et al. 2010a,b). Because goshawk nests are often reused for decades, microorganisms have the possibility to accumulate over time (Bonhoeffer et al. 1996), although we found little evidence consistent with that hypothesis. Krüger et al. (2012) showed that poor-quality territories of the goshawk were generally only occupied in years with high population density, while only core territories were occupied in years with low population density. Our findings provide limited evidence of accumulation of microorganisms across years being measurable at the level of adults or nestlings.

The findings presented here are consistent with the hypothesis that the abundance of certain bacteria, but not others is associated with the timing of breeding. Because probability of recruitment of offspring in our study population of goshawks is related to time of breeding, these results suggest a role for bacteria in determining important fitness components of goshawks and perhaps other bird species.

## Supporting Information

**Table S1.** GLMM for abundance of mesophilic bacteria, *Enterococcus* and Enterobacteriaceae in goshawk nestlings in relation to standardized laying date, age of female (years), territory occupation (no. years), sex and study year.

## References

[b1] Beaver PC, Jung RC (1985). Animal agents and vectors of human disease.

[b2] Benskin CMH, Wilson K, Jones K, Hartley IR (2009). Bacterial pathogens in wild birds: a review of the frequency and effects of infection. Biol. Rev.

[b3] Benskin CMH, Rhodes G, Pickup RW, Mainwaring MC, Wilson K, Hartley IR (2015). Life history correlates of fecal bacterial species richness in a wild population of the blue tit *Cyanistes caeruleus*. Ecol. Evol.

[b5] Bonhoeffer S, Lenski S, Ebert D (1996). The curse of the pharaoh: the evolution of virulence in pathogens with long living propagules. Proc. R. Soc. Lond. B.

[b6] Brandl HB, van Dongen WFD, Darolová A, Kristifík J, Majtan J, Hoi H (2014). Composition of bacterial assemblages in different components of reed warbler nests and a possible role of egg incubation in pathogen regulation. PLoS One.

[b8] Burfield I, van Bommel F (2004). Birds in Europe.

[b10] Cook MI, Beissinger SR, Toranzos GA, Rodriguez RA, Arendt WJ (2003). Trans-shell infection by pathogenic micro-organisms reduces the shelf life of non-incubated bird's eggs: a constraint on the onset of incubation?. Proc. R. Soc. Lond. B.

[b11] Cook MI, Beissinger SR, Toranzos GA, Rodriguez RA, Arendt WJ (2005). Microbial infection affects egg viability and incubation behavior in a tropical passerine. Behav. Ecol.

[b12] Cramp S, Simmons KEL (1980). The birds of the Western Palearctic.

[b13] Donachie SP, Foster JS, Brown MV (2007). Culture clash: challenging the dogma of microbial diversity. ISME J.

[b14] Drent RH (2006). The timing of birds' breeding seasons: the Perrins hypothesis revisited especially for migrants. Ardea.

[b15] Evans AS, Brachman PS (1998). Bacterial infections of humans.

[b16] Franz CMAP, Holzapfel WH, Stiles ME (1999). Enterococci at the crossroads of food safety?. Int. J. Food Microbiol.

[b17] García-Longoria Batanete L, Garamszegi LZ, Møller AP (2014). Host escape behavior and blood parasite infections in birds. Behav. Ecol.

[b18] Herfindal I, van de Pol M, Nielsen JT, Sæther B-E, Møller AP (2015). Climatic conditions cause complex patterns of covariation between demographic traits in a long-lived raptor. J. Anim. Ecol.

[b19] Houston CS, Saunders JR, Crawford RD (1997). Aerobic bacterial flora of addled raptor eggs in Saskatchewan. J. Wildl. Dis.

[b20] Hubálek Z (2004). An annotated checklist of pathogenic micro-organisms associated with migratory birds. J. Wildl. Dis.

[b21] Hubálek Z, Halouzka J (1996). Arthropod-borne viruses of vertebrates in Europe. Acta Sci. Nat. Brno.

[b22] Hudson PJ (1986). The effect of a parasitic nematode on the breeding production of red grouse. J. Anim. Ecol.

[b24] Kenward R (2006). The Goshawk.

[b25] Krieg NR, Holt JG (1984). Bergey's manual of systematic bacteriology.

[b26] Krüger O, Chakarov N, Nielsen JT, Looft V, Grünkorn T, Struwe-Juhl B (2012). Population regulation by habitat heterogeneity or individual adjustment?. J. Anim. Ecol.

[b27] Kühlapfel O, Brune J (1995). Die Mauserfeder als Hilfsmittel zur Altersbestimmung und Individualerkennung von Habichten (*Accipiter gentilis*. Charadrius.

[b28] Lee WY, Lee KH, Chun J, Choe JC, Jablonski PG, Lee SI (2013). Comparison of a culture-based and a PCR-based methods for estimating bacterial abundance on eggshells, with comments on statistical analyses. J. Field Ornithol.

[b31] Mennerat A, Mirleau P, Blondel J, Perret P, Lambrechts M, Heeb P (2009). Aromatic plants in nests of the blue tit *Cyanistes caeruleus* protect chicks from bacteria. Oecologia.

[b32] Møller AP, Erritzøe J (1996). Parasite virulence and host immune defence: host immune response is related to nest re-use in birds. Evolution.

[b33] Møller AP, Erritzøe J (2000). Predation against birds with low immunocompetence. Oecologia.

[b34] Møller AP, Ibáñez-Álamo JD (2012). Escape behaviour of birds provides evidence of predation being involved in urbanization. Anim. Behav.

[b35] Møller AP, Nielsen JT (2007). Malaria and risk of predation: a comparative study of birds. Ecology.

[b36] Møller AP, Erritzøe J, Nielsen JT (2010a). Predators and micro-organisms of prey: goshawks prefer prey with small uropygial glands. Funct. Ecol.

[b37] Møller AP, Flensted-Jensen E, Klarborg K, Mardal W, Nielsen JT (2010b). Climate change affects the duration of the reproductive season in birds. J. Anim. Ecol.

[b38] Møller AP, Christiansen SS, Mousseau TA (2011a). Sexual signals, risk of predation and escape behavior. Behav. Ecol.

[b39] Møller AP, Garamszegi LZ, Peralta-Sánchez JM, Soler JJ (2011b). Migratory divides and their consequences for dispersal, population size and parasite-host interactions. J. Evol. Biol.

[b40] Møller AP, Peralta-Sánchez JM, Nielsen JT, López-Hernández E, Soler JJ (2012). Goshawk prey have more bacteria than non-prey. J. Anim. Ecol.

[b41] Møller AP, Flensted-Jensen E, Mardal W, Soler JJ (2013). Host-parasite relationship between colonial terns and bacteria is modified by a mutualism with a plant having antibacterial defenses. Oecologia.

[b42] Moreno J, Briones V, Merino S, Ballesteros C, Sanz JJ, Tomás G (2003). Beneficial effects of cloacal bacteria on growth and fledging size in nestling pied flycatchers (*Ficedula hypoleuca*) in Spain. Auk.

[b43] Murray DL, Cary JR, Keith LB (1997). Interactive effects of sublethal nematodes and nutritional status on snowshoe hare vulnerability to predation. J. Anim. Ecol.

[b44] Navarro C, Møller AP, Marzal A, de Lope F (2004). Predation risk, host immune response and parasitism. Behav. Ecol.

[b45] Nielsen JT, Drachmann J (2003). Age-dependent reproductive performance in Northern Goshawks *Accipiter gentilis*. The Ibis.

[b46] Nielsen JT, Møller AP (2006). Effects of food abundance, density and climate change on reproduction in the sparrowhawk *Accipiter nisus*. Oecologia.

[b47] Opdam P, Müskens G (1976). Use of shed feathers in population studies of *Accipiter* hawks (Aves, Accipitriformes, Accipitridae). Beaufortia.

[b48] Packer C, Holt RD, Hudson PJ, Lafferty KD, Dobson AP (2003). Keeping the herds healthy and alert: implications of predator control for infectious disease. Ecol. Lett.

[b49] Peralta-Sánchez JM, Møller AP, Martín-Platero AM, Soler JJ (2010). Number and colour composition of nest lining feathers predict eggshell bacterial community in barn swallow nests: an experimental study. Funct. Ecol.

[b50] Peralta-Sánchez JM, Martín-Vivaldi M, Martín-Platero AM, Martínez-Bueno M, Oñate M, Ruiz-Rodríguez M (2012). Avian life history traits influence eggshell bacterial loads: a comparative analysis. The Ibis.

[b51] Perrins CM (1970). Timing of birds breeding seasons. The Ibis.

[b52] Perrins CM (1991). Tits and their caterpillar food-supply. The Ibis.

[b501] Salyers AA, Whitt DD (2002). Bacterial pathogenesis.

[b58] Singleton DR, Harper RG (1998). Bacteria in old house wren nests. J. Field Ornithol.

[b59] Soler JJ, Martín-Vivaldi M, Ruiz-Rodríguez M, Valdivia E, Martín-Platero AM, Martínez-Bueno M (2008). Symbiotic association between hoopoes and antibiotic-producing bacteria that live in their uropygial gland. Funct. Ecol.

[b60] Soler JJ, Martín-Vivaldi M, Peralta-Sánchez JM, Ruiz-Rodríguez M (2010). Antibiotic-producing bacteria as a possible defence of birds against pathogenic microorganisms. Open Ornithol. J.

[b61] Soler JJ, Peralta-Sanchez JM, Flensted-Jensen E, Martin-Platero AM, Møller AP (2011a). Innate humoural immunity is related to eggshell bacterial load of European birds: a comparative analysis. Naturwissenschaften.

[b62] Soler JJ, Peralta-Sánchez JM, Martínez Bueno M, Martín-Vivaldi M, Martín-Gálvez D, Vela AI (2011b). Brood parasitism is associated with increased bacterial contamination of host eggs: bacterial loads of host and parasitic eggs. Biol. J. Linn. Soc.

[b63] Soler JJ, Peralta-Sánchez JM, Martín-Platero AM, Martín-Vivaldi M, Martínez-Bueno M, Møller AP (2012a). The evolution of size of the uropygial gland: mutualistic feather mites and uropygial secretion reduce bacterial loads of eggshells and hatching failures of European birds. J. Evol. Biol.

[b64] Soler JJ, Peralta-Sánchez JM, Martín-Vivaldi M, Martín-Platero AM, Flensted-Jensen E, Møller AP (2012b). Cognitive skills and bacterial load: comparative evidence of costs of cognitive proficiency in birds. Naturwissenschaften.

[b65] Starck JM, Ricklefs RE (1998). Avian growth and development. Evolution within the altricial-precocial spectrum.

[b66] Stolp H (1988). Microbial ecology: organisms, habitats, activities.

[b67] Strauss JH, Strauss EG (2002). Viruses and human disease.

[b68] Temple SA (1987). Do predators always capture substandard individuals disproportionately from prey populations?. Ecology.

[b70] Wimberger PH (1984). The use of green plant material in bird nests to avoid ectoparasites. Auk.

