# Supplementary material for: Pathogenic bacteria and timing of laying
Source: Ecol Evol. 2015 Mar 23;5(8):1676–85. doi: 10.1002/ece3.1473 (PMC4409415; doi:10.1002/ece3.1473)
Supplement: Supplementary file 1 [file ece30005-1676-sd1.doc]

**Table S1.** GLMM for abundance of mesophilic bacteria, *Enterococcus* and Enterobacteriaceae in goshawk nestlings in relation to standardized laying date, age of female (years), territory occupation (no. years), sex and study year. In the first model the random effect of year had a variance component of 3.056, 95% CI = -5.573 to 11.685, accounting for 58.43% of the total variance, while the random effect of locality had a variance component of 0.330, 95% CI = -0.159 to 0.837, accounting for 6.48% of the variance. The model accounted for 56% of the variance. In the second model the random effect of year had a variance component of -0.003, 95% CI = -0.033 to 0.027, accounting for 0% of the total variance, while the random effect of locality had a variance component of 0.057, 95% CI = -0.076 to 0.190, accounting for 11.37% of the variance. The model accounted for 21% of the variance. In the third model the random effect of year had a variance component of 5.168, 95% CI = -9.363 to 19.670, accounting for 65.58% of the total variance, while the random effect of locality had a variance component of 0.677, 95% CI = 0.012 to 1.342, accounting for 8.59% of the variance. The model accounted for 69% of the variance. None of the effects were statistically significant.

| Effect | *F* | d.f. | *P* | Estimate (SE) |
| --- | --- | --- | --- | --- |
| **Mesophilic bacteria** |  |  |  |  |
| Intercept | 9.18 | 1, 1.814 | 0.11 | 4.422 (1.459) |
| Laying date | 0.96 | 1, 45.69 | 0.33 | 0.033 (0.034) |
| Age of female | 0.52 | 1, 31.79 | 0.48 | -0.037 (0.051) |
| No. years occupied | 0.38 | 1, 34.83 | 0.54 | 0.010 (0.016) |
| Laying date x Year | 0.04 | 1, 49.35 | 0.85 | -0.006 (0.032) |
|  |  |  |  |  |
| ***Enterococcus*** |  |  |  |  |
| Intercept | 0.01 | 1, 6.831 | 0.98 | 0.015 (0.454) |
| Laying date | 1.51 | 1, 6.667 | 0.26 | 0.026 (0.021) |
| Age of female | 0.00 | 1, 20.11 | 0.99 | -0.0001 (0.0257) |
| No. years occupied | 0.00 | 1, 22.26 | 0.99 | -0.0001 (0.0078) |
| Laying date x Year | 0.70 | 1, 25.88 | 0.41 | 0.014 (0.017) |
|  |  |  |  |  |
| **Enterobacteriaceae** |  |  |  |  |
| Intercept | 2.34 | 1, 1.663 | 0.29 | 2.821 (1.847) |
| Laying date | 1.09 | 1, 50.38 | 0.30 | 0.041 (0.039) |
| Age of female | 0.56 | 1, 37.48 | 0.46 | 0.046 (0.061) |
| No. years occupied | 0.68 | 1, 42.43 | 0.41 | 0.015 (0.019) |
| Laying date x Year | 0.01 | 1, 53.67 | 0.94 | 0.003 (0.037) |
